# Supplementary material for: Bevacizumab or PARP-Inhibitors Maintenance Therapy for Platinum-Sensitive Recurrent Ovarian Cancer: A Network Meta-Analysis
Source: Int J Mol Sci. 2020 May 27;21(11):3805. doi: 10.3390/ijms21113805 (PMC7312982; doi:10.3390/ijms21113805)
Supplement: Supplementary file 1 [file ijms-21-03805-s001.pdf]

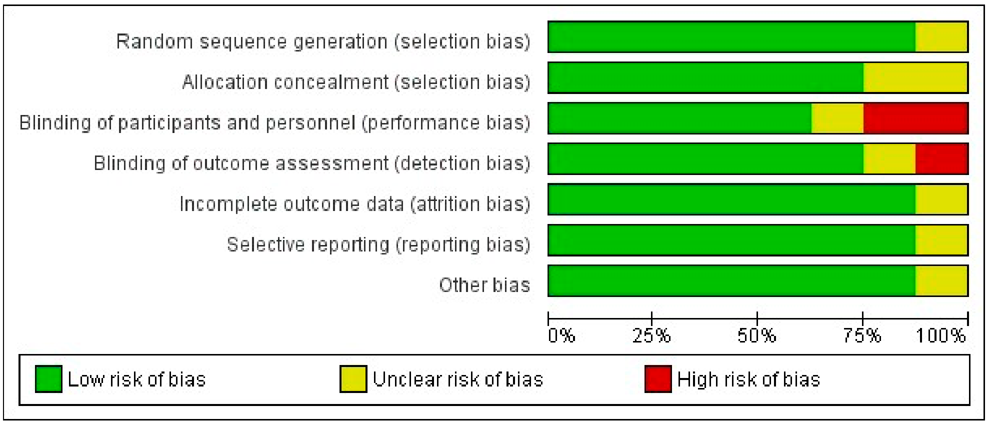
Random sequence generation (selection bias)
Allocation concealment (selection bias)
Blinding of participants and personnel (performance bias)
Blinding of outcome assessment (detection bias)
Incomplete outcome data (attrition bias)
Selective reporting (reporting bias)
Other bias
0%
25%
50%
75%
100%
Low risk of bias
Unclear risk of bias
High risk of bias

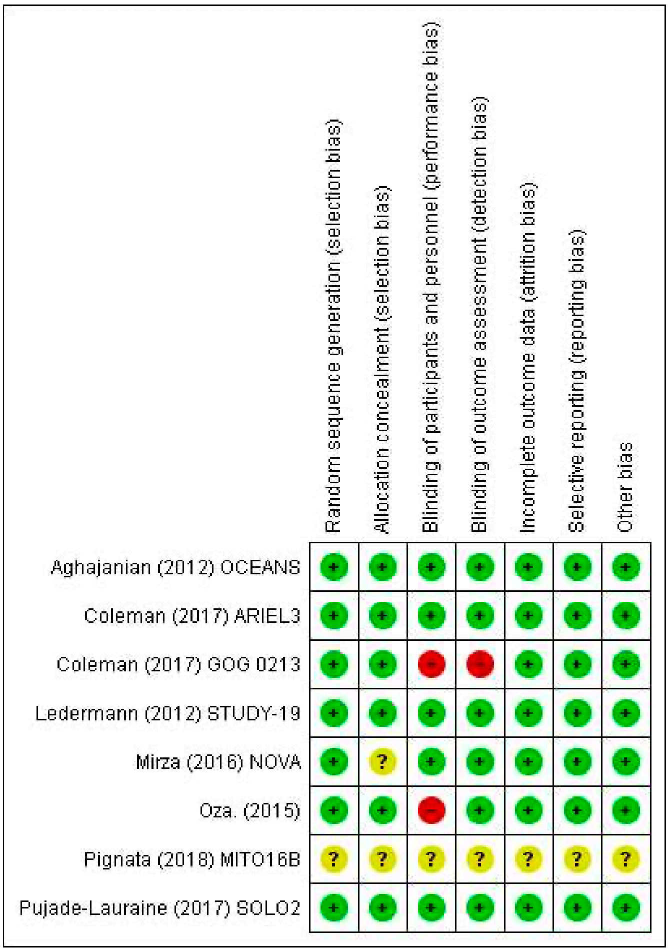
Random sequence generation (selection bias)
Allocation concealment (selection bias)
Blinding of participants and personnel (performance bias)
Blinding of outcome assessment (detection bias)
Incomplete outcome data (attrition bias)
Selective reporting (reporting bias)
Other bias
Aghajanian (2012) OCEANS
Coleman (2017) ARIEL3
Coleman (2017) GOG 0213
Ledermann (2012) STUDY-19
Mirza (2016) NOVA
Oza. (2015)
Pignata (2018) MITO16B
Pujade-Lauraine (2017) SOLO2
